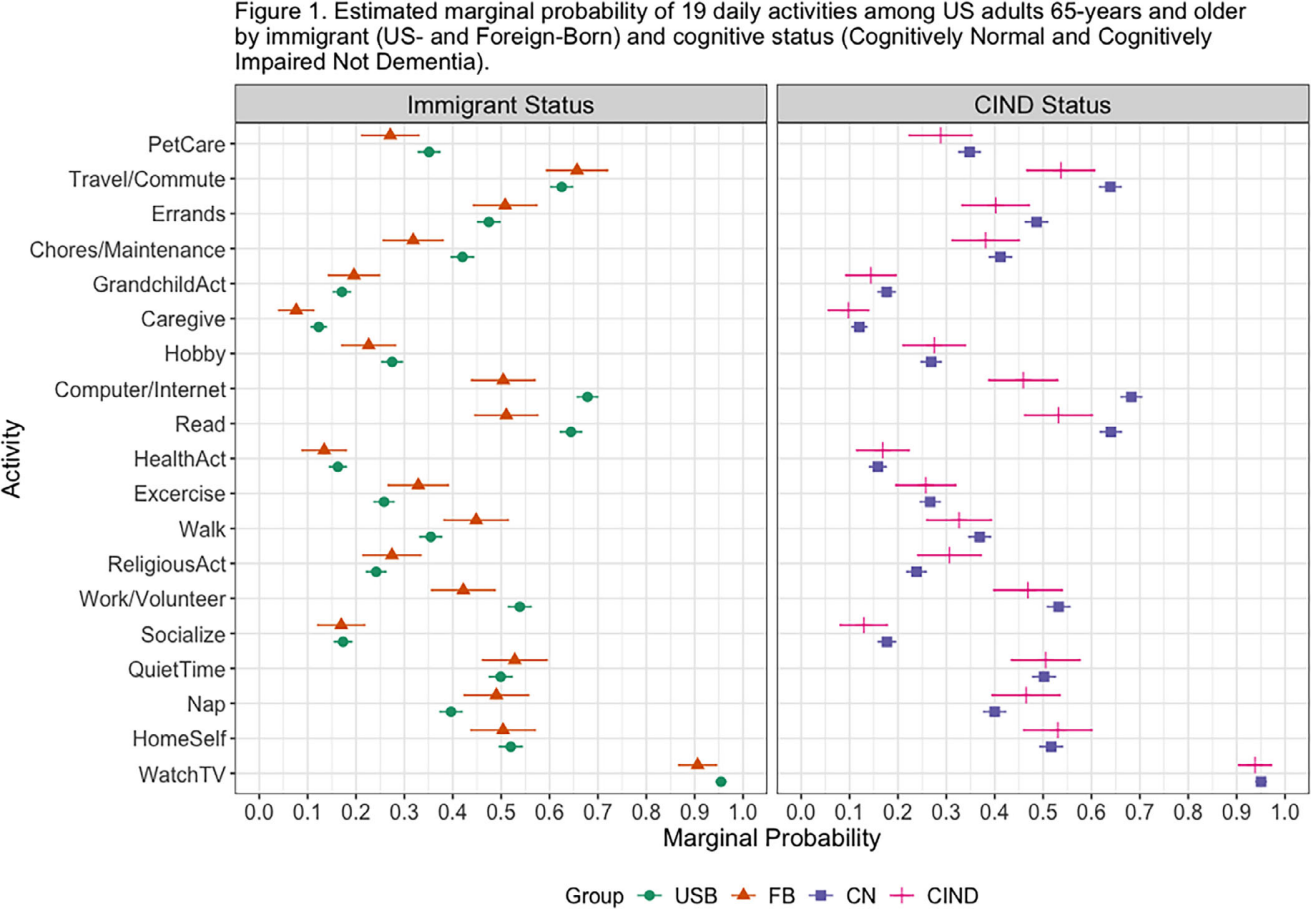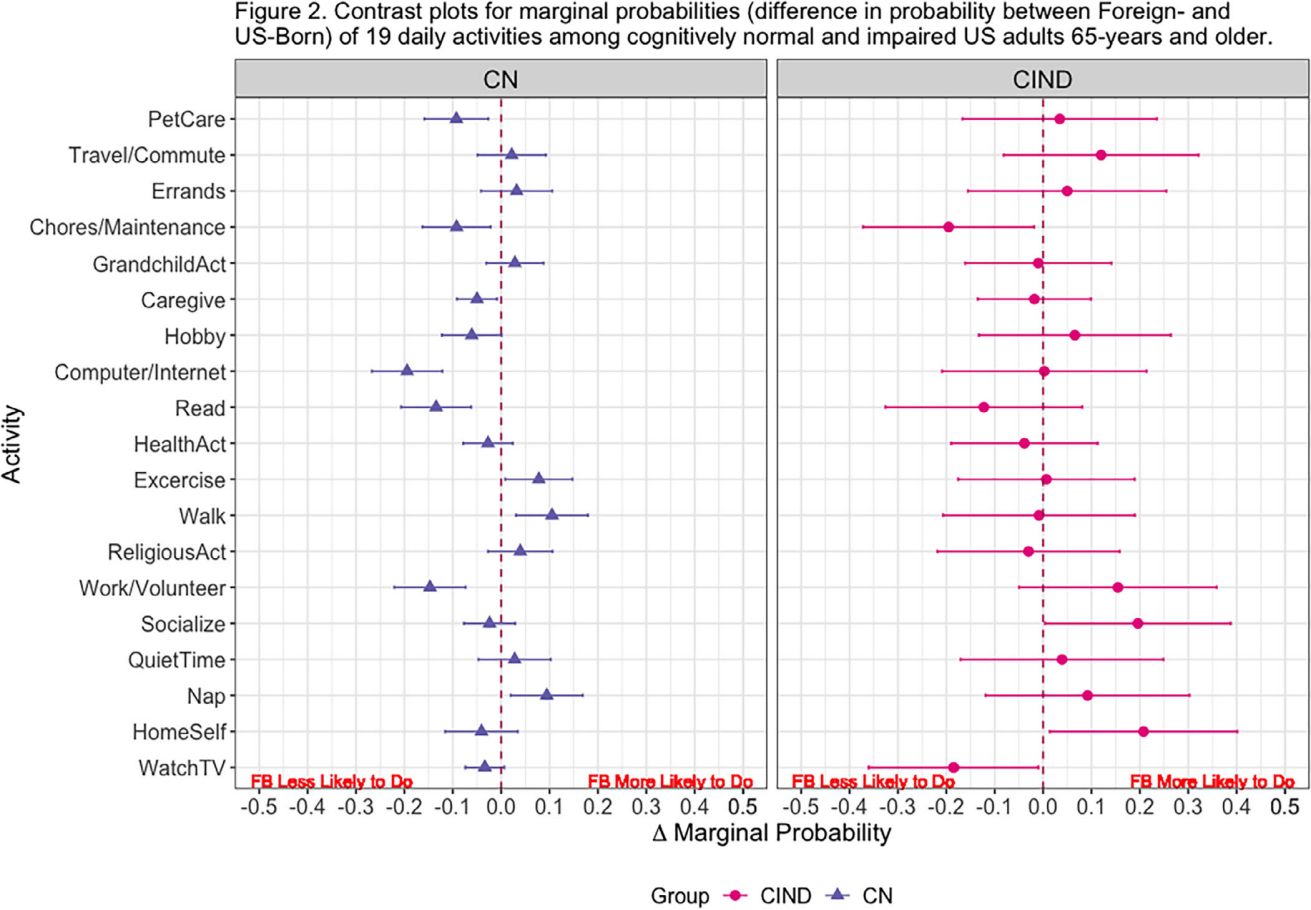# Variability in prevalent and health relevant daily activities among US‐ and Foreign‐born older adults with normal and impaired cognitive function: results from the Health and Retirement Study

**DOI:** 10.1002/alz70860_105104

**Published:** 2025-12-23

**Authors:** Wassim Tarraf, Kristine J Ajrouch, Toni C Antonucci, Simon Brauer, Laura B. Zahodne

**Affiliations:** ^1^ Wayne State University, Detroit, MI, USA; ^2^ University of Michigan, Ann Arbor, MI, USA

## Abstract

**Background:**

Stimulating activities can enhance well‐being across the lifespan as well as foster healthy physical and cognitive aging. Recommendations regarding changes to, or adoptions of, routine daily engagements can lead to low burden interventions to improve cognitive health and delay onset of cognitive impairment. Importantly, activity engagement can be influenced by sociocultural factors and as such any recommendations should be adapted to the effects of such influences. We examine patterns of daily activities engagement (based on responses to queries regarding 19 “prevalent and health relevant” activities that a respondent did the day before completing the questionnaire) among US‐born and Foreign‐born (immigrant) older adults (65+ years). We then test differences in these activities across immigrant groups in cognitively normal (CN) and cognitively impaired but not dementia (CIND) individuals.

**Method:**

We use data from *n* = 1,994 adults 65+ years with normal cognitive function in 2018 (mean age: 74‐years; 56% female) who either maintained normal cognitive status or transitioned to CIND (Langa‐Weir‐Classification) by 2020 and completed a time use questionnaire through the leave behind psychosocial and lifestyle module. We fit a series of logistic regression to examine engagement in each activity (0=No; 1=Yes) as a function of immigrant status (0=US‐born; 1=Foreign‐born), cognitive status (0=CN, 1=CIND), and their interaction, and adjusted for age, sex/gender, and a count of comorbid conditions (range=0‐8).

**Result:**

Both immigrant and cognitive status associated differently with how older adults engage in the considered activities (Figure 1). The difference between US‐Born and Foreign‐Born older adults was more evident in cognitively normal individuals but less so (with four exceptions) among those that transitioned to CIND (Figure 2). Differences were not influenced by covariate adjustment.

**Conclusion:**

US‐ and Foreign‐born older adults with healthy cognition, on average, spend time doing different daily activities. These decisions are likely influenced by sociocultural factors. Yet, their differences are reduced due to cognitive impairment providing evidence for common constraining effects of declining cognitive health across groups. Follow‐up research will validate these results with additional data (to overcome the potential confounding effects of COVID‐19) and focus on identifying typologies of activities that predict distal cognitive outcomes such as cognitive decline and impairment.